# Assessment of Barriers and Challenges in Headache Education Among Neurology Residents in Saudi Arabia

**DOI:** 10.7759/cureus.38328

**Published:** 2023-04-30

**Authors:** Abdullah A Tawakul, Sarah S Aldharman, Nouf M Al-Rabiah, Gutaybah S Alqarni, Abdulrahman A Albalawi, Omar M Alhussaini, Nawaf F Alhazmi, Abdullah R Alharbi, Omar Babateen

**Affiliations:** 1 Department of Internal Medicine, Umm Al-Qura University, Makkah, SAU; 2 College of Medicine, King Saud Bin Abdulaziz University for Health Sciences, Riyadh, SAU; 3 College of Medicine, King Faisal University, Al-Ahsa, SAU; 4 College of Medicine, University of Jeddah, Jeddah, SAU; 5 College of Medicine, University of Tabuk, Tabuk, SAU; 6 College of Medicine, Alrayan Medical Colleges, Medina, SAU; 7 College of Medicine, Umm Al-Qura University, Makkah, SAU; 8 Department of Physiology, Umm Al-Qura University, Makkah, SAU

**Keywords:** saudi arabia, neurology, residency, barrier, education, headache

## Abstract

Background

Headache problems are among the most common medical conditions. There are major gaps in understanding headaches among healthcare practitioners. This study aimed to determine challenges and barriers to headache training among neurology residents in Saudi Arabia.

Methods

A cross-sectional questionnaire-based study was conducted in Saudi Arabia. The target population was all Saudi neurology residents who were currently registered with the Saudi Commission for Health Specialties (SCHS). Statistical analysis was conducted using RStudio (R version 4.1.1). A chi-squared test is used for categorical variables whenever applicable. The statistical differences for continuous variables were assessed using a Wilcoxon rank sum test.

Results

A total of 227 respondents were included. More than half of the residents were male (56.8%). Large proportions of residents self-rated their knowledge as good for migraines (62.6%) and tension-type headaches (60.4%). The most experienced challenges included difficult diagnosis (30.4%) and treatment difficulties due to comorbidities (19.8%). The most stated barriers to optimal treatment of headache patients were the existence of challenges in collaboration between patient and therapist (24.7%). Most residents rely on the use of the International Classification of Headache Disorders (ICHD) in diagnosing and managing headache patients instead of the Saudi guidelines. The most reported reason for headache referral was suspicion of an underlying serious disease (31.7%). The most recommended non-pharmacological interventions were exercise (15.9%).

Conclusion

We found that residents reported the diagnosis of headache as the most challenging barrier. The overuse of analgesics played a role in causing the headache. The most often cited barrier to providing headache patients with the best possible care was difficulties in patient-therapist collaboration. Ongoing headache education and comprehensive academic training are recommended to enhance knowledge during neurology residency training and offer competent care for their patients.

## Introduction

According to the Global Burden of Disease (GBD) study, headache disorders are one of the most prevalent and disabling conditions worldwide [1]. Despite this fact, there are grave shortcomings in healthcare providers’ awareness that are observed on a global scale [2]. Many countries don’t regard headaches as a condition requiring healthcare, and their priority is low in all countries [3]. Along with cultural and socioeconomic factors, educational deficiency among healthcare providers is a barrier to providing patients with proper headache care [4]. Although headache disorders are one of the top 10 causes of death and disability in Saudi Arabia, most participants in a study conducted in Saudi Arabia on headache disorders concur that there is a need for more education for headache patients, which reflects the extent of the lack of education about headache, despite its importance [5,6]. 

In a study conducted in Denmark as a national cross-sectional survey among residents in neurology training, the author found various gaps and barriers in formal headache education and training among neurology residents in the management of headache disorders related to challenges in diagnosis. As it will lead to inadequate care, they also discovered that a better understanding of headache education would probably lead to better clinical results. The questionnaire asked questions about participant demographics, understanding of and barriers to headache problems, usage of guidelines and diagnostic tools, medication overuse, and non-pharmacological interventions. Approximately half of those surveyed stated that diagnosing and treating headache patients is difficult. This is especially concerning given that the majority of consultations are estimated to be 11%-20% headache-related. Because most headache disorders lack biomarkers or diagnostic tests, diagnosis is based solely on the patient's medical history. While headache diaries, in general, are used for diagnosis and outcome assessment. The expectation is that as experience accumulates, residents will use and understand diagnostic criteria more consistently [7]. Another study was conducted in the United States of America and contained a survey sent to family medicine and neurology residents. According to the survey's findings, the majority of family practice program directors (87.5%) thought their residency program's headache education was sufficient, while 95.4% of neurology practice program directors that their residents were adequately trained in headaches. Interestingly, it was stated program directors thought new neurology and family medicine residents had inadequate knowledge about headaches upon entering the program (68%, and 67.5%, respectively). It's interesting to note that 20% of neurology programs are intended to increase headache treatment training. Neurology and family practice residency program directors believe new residents are weak in headache knowledge upon entering the program [8].

A study was conducted to evaluate the state of headache medicine practice and teaching in American medical schools, and surveys were sent to departmental chairs and residency training directors. Researchers were able to gather information from 95 (71.4%) of the programs offering training for residents in neurology. The author provides a straightforward description of a situation that needs more research. Individual neurology departments will have to deal with a gap between the lack of training in headache medicine and the belief in its validity as an important disease management area [9]. Many studies have assessed headache medicine education in neurology residency programs. In Norway, a study was done to assess headache knowledge among 17 hospitals with 143 neurology resident participants; the result showed they had moderate knowledge at best [10]. Another study published in 2017 assessed headache interest, and the result suggests that there are strong associations between under-emphasis on headache education within neurology residency training and a lack of neurologists interested in headache medicine [11]. In a study done in 2018 in Houston to evaluate the proportion of headache-related grand rounds, the results showed that headache is adequately represented in grand rounds. However, it makes up the lowest proportion of grand rounds lectures of all core Accreditation Council for Graduate Medical Education [ACGME] milestones [12]. It is expected that neurology residents at all levels obtain knowledge about headache disorders through clinical experience in combination with self-study. Efforts should be made to pinpoint major gaps in knowledge among healthcare professionals to facilitate better educational policies in headache training. This study aimed to determine the challenges and barriers to headache training among neurology residents in Saudi Arabia.

## Materials and methods

This descriptive cross-sectional questionnaire-based study was conducted in Saudi Arabia between August 2022 and November 2022. The target subjects were all Saudi neurology residents who were currently registered with the Saudi Commission for Health Specialties (SCHS) from different regions. The study was conducted through a self-administered questionnaire distributed on different online platforms. All responses were collected and exported into a Microsoft Excel (Redmond, USA) spreadsheet file using Google Docs tools for processing and analyzing information. The data were analyzed using RStudio (R version 4.1.1).

Sample size and sampling technique

OpenEpi® version 3.0 software was employed to calculate the sample size. The representative sample size required was 80, with a margin error determined as 5% and a confidence level determined as 95%, and the population determined as approximately 100 [13]. We aimed to obtain more than the calculated sample size to overcome any exclusions. Therefore, non-probability convenience sampling techniques have been employed.

Inclusion criteria and exclusion criteria

The study's eligibility criteria included all Saudi neurology residents who were currently registered with the Saudi Commission for Health Specialties (SCHS) from different regions of Saudi Arabia. The neurology residency program is divided into two parts: the introductory program (1st-year residents) and the main program (2nd, 3rd, 4th, and 5th-year residents). Pediatric neurology was excluded from this survey as we only invited residents in adult neurological residency since pediatric neurology has a separate residency training. Participants who did not complete the questionnaire or did not agree to participate were excluded.

Data collection instruments and procedures

We employed a self‐administered questionnaire that was adapted from a previous study on comparable objectives [7]. The questionnaire consisted of questions on participant demographics, knowledge of and challenges to headache disorders, and utilization of guidelines and diagnostic tools. It also covered areas of specific importance, such as medication overuse and non-pharmacological therapies. The questionnaire was distributed in the English language. The questionnaire was distributed electronically using Google Forms on different social media platforms such as WhatsApp, Twitter, and Telegram.

All information was kept private and used for scientific research. Participation in this study was voluntary and optional, with informed consent provided to all participants on the first page before filling out the questionnaire. The ethical approval of the study was obtained before initiating it. The ethical approval was obtained from the Biomedical Ethics Committee of Umm Al-Qura University (approval number: HAPO-02-K-012-2022-09-1190).

Statistical analysis

After distributing the questionnaires, they were checked for completeness and any missing information. The collected data was first entered into a Microsoft Excel (Redmond, USA) file. Statistical analysis was conducted using RStudio (R version 4.1.1., Posit, Boston, MA, USA). We used frequencies and percentages to present categorical data and mean ± standard deviation (SD) to express continuous variables. Between-group differences between residents of introductory and main programs were assessed using a Fisher's exact test or Pearson's Chi-squared test for categorical variables whenever applicable. The statistical differences for continuous variables were assessed using a Wilcoxon rank sum test. Statistical significance was deemed at p < 0.05.

## Results

Demographic characteristics of neurology residents 

We initially collected 267 responses on the online platform. However, 40 records were excluded because the respondents were not neurology residents. Therefore, the data of 227 respondents were analyzed. More than half of the residents were male (56.8%). Residents of the Central and Eastern regions represented 40.5% and 27.8% of the sample, respectively. Neurology residents in the introductory program (R1) constituted 28.6% of the participants, whereas 71.4% of them were allocated to the main program. The majority of them (74.9%) received prior headache training. More details on demographics are shown in Table 1.

**Table 1:** Demographic characteristics of neurology residents

Parameter	Category	N (%)
Gender	Male	129 (56.8%)
	Female	98 (43.2%)
Residency stage	R1	65 (28.6%)
	R2	65 (28.6%)
	R3	63 (27.8%)
	R4	27 (11.9%)
	R5	7 (3.1%)
Region of neurology residency training	Central	92 (40.5%)
	Eastern	63 (27.8%)
	Western	56 (24.7%)
	Southern	16 (7.0%)
Prior headache education/training	Yes	170 (74.9%)

Self-reported knowledge, barriers, and challenges in headache disorders

**Figure 1:**
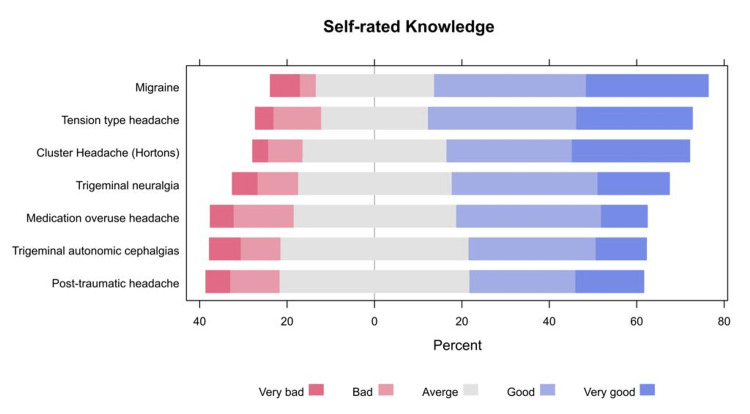
The percentages of participants’ responses to their items related to their self-reported knowledge about headache disorders

Large proportions of residents self-rated their knowledge as good or very good for migraine (62.6%), tension-type headache (60.4%), and cluster headache (55.5%), whereas less than a half of residents self-rated their knowledge as good or very good for trigeminal headache (39.6%), autonomic cephalgias (40.5%), and medication overuse headache (43.6%, Figure 1).

**Figure 2:**
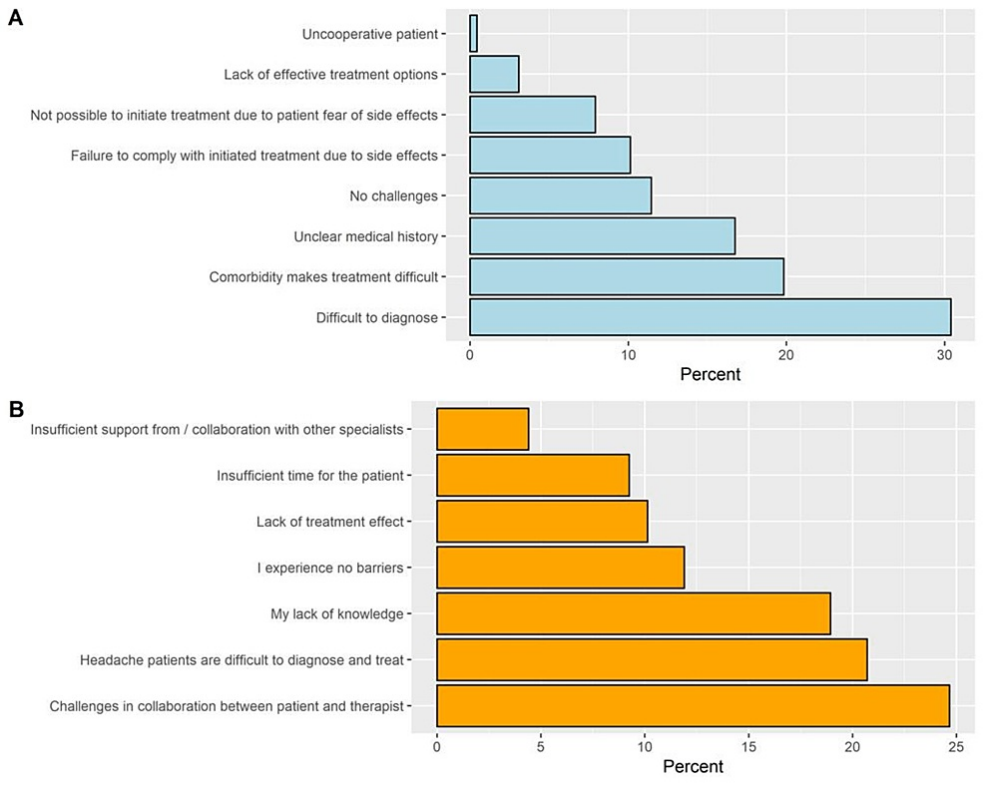
The percentages of participants’ responses to the challenges experienced in relation to headache patients (A) and the barriers to optimal treatment (B).

The most commonly experienced challenges by the residents included difficult diagnosis (30.4%), treatment difficulties due to comorbidities (19.8%), and unclear medical histories, whereas 11.5% of residents experienced no challenges (Figure 2A). The most frequently reported barriers to optimal treatment of headache patients were the existence of challenges in collaboration between patient and therapist (24.7%) and difficulties in the diagnosis of headache patients (20.7%, Figure 2B).

Use of guidelines, classification, and tools for diagnosis and outcome assessment

In general, the score using the International Classification of Headache Disorders (ICHD) was relatively higher than that of using the Saudi guidelines for diagnosis and management of headaches (3.53 ± 1.14 vs. 3.38 ± 1.28, respectively). Utilization of the Saudi guidelines was significantly higher among residents of the introductory program than those of the main program (3.74 ± 1.19 vs. 3.23 ± 1.29, respectively, p = 0.007); however, there was no significant difference in the utilization of the ICHD guidelines between residents of the introductory program and those of the main program. More details about the use of classification schemes and diagnostic tools are demonstrated in Table 2.

**Table 2:** Use of guidelines, classification and tools for diagnosis and outcome assessment *The variable was coded as 1=Never, 2=Less than half of the time, 3=At least half of the time and 4=Always Other variables were coded from 1=Never to 5=Always

Parameter	Overall, N = 227	Introductory program, N = 65	Main program, N = 162	p-value
Using the Saudi guidelines for diagnosis and management of headache	3.38 ± 1.28	3.74 ± 1.19	3.23 ± 1.29	0.007
Using the International Classification of Headache Disorders (ICHD)	3.53 ± 1.14	3.49 ± 1.21	3.55 ± 1.11	0.990
Asking patients to complete a headache diary to make a diagnosis	3.26 ± 1.17	3.08 ± 1.25	3.33 ± 1.13	0.256
Using a headache calendar for follow-up on treatment	3.28 ± 1.14	3.14 ± 1.21	3.33 ± 1.11	0.363
Asking the patients if their headache affects their quality of life*	2.85 ± 1.03	3.00 ± 1.06	2.78 ± 1.02	0.140

Contact and referral patterns

As shown in Table 3, the proportion of headache consultations to neurology was 30% or higher among 17.4% of residents. The proportion of residents in the main program with >40% headache consultations was significantly higher than their counterparts in the introductory program (6.2% vs. 1.5%, p = 0.014). Only 19.8% of residents indicated that they were consulted for professional advice from general practitioners regarding headache patients, whereas 44.3% of them revealed that their collaboration with GPs was good to very good concerning the referred patients with headaches. Of note, only 8.3% of residents referred >30% of headache patients to specialized headache centers, and the most commonly reported reasons for referral to neurology were a suspicion of an underlying serious disease (31.7%) and diagnostic uncertainty (30.8%). Additionally, 15% of residents indicated that the waiting time for patients referred for specialized treatment was long or unacceptably long, while 18.1% of residents stated that the referral was helpful to a large or great extent.

**Table 3:** Contact and referral patterns *the variable has eight missing records

Parameter	Category	Overall, N = 227	Introductory program, N = 65	Main program, N = 162	p-value
What proportion of your patient consultations are related to headache?	None	2 (0.9%)	1 (1.5%)	1 (0.6%)	0.014
	1-10%	42 (18.5%)	21 (32.3%)	21 (13.0%)	
	11-20%	72 (31.7%)	19 (29.2%)	53 (32.7%)	
	21-30%	71 (31.3%)	15 (23.1%)	56 (34.6%)	
	31-40%	29 (12.8%)	8 (12.3%)	21 (13.0%)	
	>40%	11 (4.8%)	1 (1.5%)	10 (6.2%)	
Do general practitioners contact you for professional advice regarding headache patients?	Never	18 (7.9%)	7 (10.8%)	11 (6.8%)	0.576
	Rarely	68 (30.0%)	17 (26.2%)	51 (31.5%)	
	Once in a while	96 (42.3%)	31 (47.7%)	65 (40.1%)	
	Frequently	33 (14.5%)	7 (10.8%)	26 (16.0%)	
	Very frequently	12 (5.3%)	3 (4.6%)	9 (5.6%)	
How would you describe your collaboration with General Practitioners concerning referred headache patients?*	Non existing	2 (0.9%)	0 (0.0%)	2 (1.3%)	0.909
	Very bad/Non existing	0 (0.0%)	0 (0.0%)	0 (0.0%)	
	Bad	22 (10.0%)	6 (9.7%)	16 (10.2%)	
	Neither good nor bad	98 (44.7%)	31 (50.0%)	67 (42.7%)	
	Good	78 (35.6%)	20 (32.3%)	58 (36.9%)	
	Very good	19 (8.7%)	5 (8.1%)	14 (8.9%)	
What proportion of your headache patients do you refer to treatment at specialized headache centers?	I never refer patients	14 (6.2%)	5 (7.7%)	9 (5.6%)	0.059
	1-10%	67 (29.5%)	26 (40.0%)	41 (25.3%)	
	11-20%	62 (27.3%)	17 (26.2%)	45 (27.8%)	
	21-30%	65 (28.6%)	13 (20.0%)	52 (32.1%)	
	31-40%	16 (7.0%)	2 (3.1%)	14 (8.6%)	
	>40%	3 (1.3%)	2 (3.1%)	1 (0.6%)	
What is your most common reason for referring headache patients?	None	1 (0.4%)	0 (0.0%)	1 (0.6%)	0.516
	Suspicion of serious underlying cause	72 (31.7%)	19 (29.2%)	53 (32.7%)	
	Desire/expectation of the patient	33 (14.5%)	11 (16.9%)	22 (13.6%)	
	Diagnostic uncertainty	70 (30.8%)	24 (36.9%)	46 (28.4%)	
	Lack of treatment effect	51 (22.5%)	11 (16.9%)	40 (24.7%)	
How do you consider the wait time for patients you refer to specialized treatment?	Short	31 (13.7%)	14 (21.5%)	17 (10.5%)	0.083
	Acceptable	162 (71.4%)	43 (66.2%)	119 (73.5%)	
	Long	27 (11.9%)	5 (7.7%)	22 (13.6%)	
	Unacceptable long	7 (3.1%)	3 (4.6%)	4 (2.5%)	
To what extent do you find it helpful for patients to be referred?	Not at all	16 (7.0%)	4 (6.2%)	12 (7.4%)	0.732
	To a small extent	58 (25.6%)	16 (24.6%)	42 (25.9%)	
	To some extent	89 (39.2%)	25 (38.5%)	64 (39.5%)	
	To a large extent	36 (15.9%)	9 (13.8%)	27 (16.7%)	
	To a great extent	5 (2.2%)	1 (1.5%)	4 (2.5%)	
	Do not know	23 (10.1%)	10 (15.4%)	13 (8.0%)	
Parameter	Category	Overall, N = 227	Introductory program, N = 65	Main program, N = 162	p-value
What proportion of your patient consultations are related to headache?	None	2 (0.9%)	1 (1.5%)	1 (0.6%)	0.014
	1-10%	42 (18.5%)	21 (32.3%)	21 (13.0%)	
	11-20%	72 (31.7%)	19 (29.2%)	53 (32.7%)	
	21-30%	71 (31.3%)	15 (23.1%)	56 (34.6%)	
	31-40%	29 (12.8%)	8 (12.3%)	21 (13.0%)	
	>40%	11 (4.8%)	1 (1.5%)	10 (6.2%)	
Do general practitioners contact you for professional advice regarding headache patients?	Never	18 (7.9%)	7 (10.8%)	11 (6.8%)	0.576
	Rarely	68 (30.0%)	17 (26.2%)	51 (31.5%)	
	Once in a while	96 (42.3%)	31 (47.7%)	65 (40.1%)	
	Frequently	33 (14.5%)	7 (10.8%)	26 (16.0%)	
	Very frequently	12 (5.3%)	3 (4.6%)	9 (5.6%)	
How would you describe your collaboration with General Practitioners concerning referred headache patients?*	Non existing	2 (0.9%)	0 (0.0%)	2 (1.3%)	0.909
	Very bad/Non existing	0 (0.0%)	0 (0.0%)	0 (0.0%)	
	Bad	22 (10.0%)	6 (9.7%)	16 (10.2%)	
	Neither good nor bad	98 (44.7%)	31 (50.0%)	67 (42.7%)	
	Good	78 (35.6%)	20 (32.3%)	58 (36.9%)	
	Very good	19 (8.7%)	5 (8.1%)	14 (8.9%)	
What proportion of your headache patients do you refer to treatment at specialized headache centers?	I never refer patients	14 (6.2%)	5 (7.7%)	9 (5.6%)	0.059
	1-10%	67 (29.5%)	26 (40.0%)	41 (25.3%)	
	11-20%	62 (27.3%)	17 (26.2%)	45 (27.8%)	
	21-30%	65 (28.6%)	13 (20.0%)	52 (32.1%)	
	31-40%	16 (7.0%)	2 (3.1%)	14 (8.6%)	
	>40%	3 (1.3%)	2 (3.1%)	1 (0.6%)	
What is your most common reason for referring headache patients?	None	1 (0.4%)	0 (0.0%)	1 (0.6%)	0.516
	Suspicion of serious underlying cause	72 (31.7%)	19 (29.2%)	53 (32.7%)	
	Desire/expectation of the patient	33 (14.5%)	11 (16.9%)	22 (13.6%)	
	Diagnostic uncertainty	70 (30.8%)	24 (36.9%)	46 (28.4%)	
	Lack of treatment effect	51 (22.5%)	11 (16.9%)	40 (24.7%)	
How do you consider the wait time for patients you refer to specialized treatment?	Short	31 (13.7%)	14 (21.5%)	17 (10.5%)	0.083
	Acceptable	162 (71.4%)	43 (66.2%)	119 (73.5%)	
	Long	27 (11.9%)	5 (7.7%)	22 (13.6%)	
	Unacceptable long	7 (3.1%)	3 (4.6%)	4 (2.5%)	
To what extent do you find it helpful for patients to be referred?	Not at all	16 (7.0%)	4 (6.2%)	12 (7.4%)	0.732
	To a small extent	58 (25.6%)	16 (24.6%)	42 (25.9%)	
	To some extent	89 (39.2%)	25 (38.5%)	64 (39.5%)	
	To a large extent	36 (15.9%)	9 (13.8%)	27 (16.7%)	
	To a great extent	5 (2.2%)	1 (1.5%)	4 (2.5%)	
	Do not know	23 (10.1%)	10 (15.4%)	13 (8.0%)	

Medication overuse for headache

Approximately one-third of residents (36.1%) found that medications were overused to some extent, whereas 14.5% found that the problem was apparent to a large or great extent. Simple analgesics could potentially cause medication overuse, as perceived by 33.5% of residents, with no significant difference in residents’ perceptions between the introductory and main programs. A total of 40 residents (17.6%) did not know about the recommended maximum use in terms of dose and duration of simple analgesics for headache patients. A significantly higher proportion of residents in the introductory program did not know about the recommended maximum use of simple analgesics for headache patients compared to residents in the main program (32.3% vs. 11.7%, p = 0.009, Table 4).

**Table 4:** Medication overuse for headache

Parameter	Category	Overall, N = 227	Introductory program, N = 65	Main program, N = 162	p-value
To what extent do you find that medication overuse headache is a problem among your headache patients?	Not at all	19 (8.4%)	5 (7.7%)	14 (8.6%)	0.036
	To a small extent	93 (41.0%)	27 (41.5%)	66 (40.7%)	
	To some extent	82 (36.1%)	18 (27.7%)	64 (39.5%)	
	To a large extent	23 (10.1%)	13 (20.0%)	10 (6.2%)	
	To a great extent	10 (4.4%)	2 (3.1%)	8 (4.9%)	
Do you know what kind of medication that can potentially cause medication overuse headache?	Migraine preventive medicine	34 (15.0%)	10 (15.4%)	24 (14.8%)	0.155
	Migraine acute medicine	44 (19.4%)	10 (15.4%)	34 (21.0%)	
	Simple analgesics	76 (33.5%)	28 (43.1%)	48 (29.6%)	
	Opioids	64 (28.2%)	13 (20.0%)	51 (31.5%)	
	Do not know	9 (4.0%)	4 (6.2%)	5 (3.1%)	
Do you know the recommended maximum use of simple analgesics for headache patients?	1 day a week	29 (12.8%)	8 (12.3%)	21 (13.0%)	0.009
	2-3 days a week	107 (47.1%)	23 (35.4%)	84 (51.9%)	
	4-5 days a week	42 (18.5%)	11 (16.9%)	31 (19.1%)	
	6 days a week	9 (4.0%)	2 (3.1%)	7 (4.3%)	
	Do not know	40 (17.6%)	21 (32.3%)	19 (11.7%)	

Non-pharmacological interventions

Almost half of the residents stated that patients seek a non-pharmacological option for headache treatment once in a while (45.4%). Only 11.5% of residents felt equipped to a large or great extent to advise patients on non-pharmacological intervention options for headaches, and a significantly higher proportion of residents in the introductory program did not feel equipped to advise patients than those in the main program (18.5% vs. 5.6%, p = 0.037). If asked by the patient, the most commonly recommended non-pharmacological interventions were exercise (15.9%) and diet (14.1%). There were no significant differences in the types of non-pharmacological interventions between the introductory and main programs except for physiotherapy recommendations (15.4% among residents of the introductory program vs. 4.3% among residents of the main program, p = 0.009, Table 5).

**Table 5:** Non-pharmacological interventions

Parameter	Category	Overall, N = 227	Introductory program, N = 65	Main program, N = 162	p-value
Do your patients seek your advice on non-pharmacological treatment options for headaches?	Never	24 (10.6%)	11 (16.9%)	13 (8.0%)	0.085
	Rarely	68 (30.0%)	20 (30.8%)	48 (29.6%)	
	Once in a while	103 (45.4%)	24 (36.9%)	79 (48.8%)	
	Frequently	20 (8.8%)	4 (6.2%)	16 (9.9%)	
	Very frequently	12 (5.3%)	6 (9.2%)	6 (3.7%)	
To what extent do you feel equipped to advise your patients on non-medical treatment options?	Not at all	21 (9.3%)	12 (18.5%)	9 (5.6%)	0.037
	To a small extent	100 (44.1%)	28 (43.1%)	72 (44.4%)	
	To some extent	80 (35.2%)	18 (27.7%)	62 (38.3%)	
	To a large extent	21 (9.3%)	5 (7.7%)	16 (9.9%)	
	To a great extent	5 (2.2%)	2 (3.1%)	3 (1.9%)	
What types of treatment would you recommend if asked by your patients?	Diet	32 (14.1%)	7 (10.8%)	25 (15.4%)	0.361
	Cranio-sacral therapy	4 (1.8%)	0 (0.0%)	4 (2.5%)	0.580
	Psychological treatment	28 (12.3%)	10 (15.4%)	18 (11.1%)	0.376
	Neurostimulation	3 (1.3%)	2 (3.1%)	1 (0.6%)	0.198
	Physiotherapy	17 (7.5%)	10 (15.4%)	7 (4.3%)	0.009
	Exercise	36 (15.9%)	9 (13.8%)	27 (16.7%)	0.599
	Reflexology	16 (7.0%)	7 (10.8%)	9 (5.6%)	0.249
	Acupuncture	18 (7.9%)	2 (3.1%)	16 (9.9%)	0.087
	Ear piercing	2 (0.9%)	0 (0.0%)	2 (1.2%)	>0.999
	I do not recommend any of these treatments	41 (18.1%)	13 (20.0%)	28 (17.3%)	0.631

Interest in neurological sub-specializations

The highest scores of interest in neurological sub-specialties were relevant to headache (2.75 ± 1.43), dementia (2.66 ± 1.32), and neuromuscular disease (2.57 ± 1.37), whereas the lowest interest scores were reported for epilepsy (2.31 ± 1.23) and multiple sclerosis (2.36 ± 1.27, Table 6).

**Table 6:** Interest in neurological sub-specializations

Parameter	Overall, N = 227	Introductory program, N = 65	Main program, N = 162	p-value
Cerebrovascular diseases	2.41 ± 1.34	2.40 ± 1.46	2.42 ± 1.29	0.685
Dementia	2.66 ± 1.32	2.82 ± 1.33	2.60 ± 1.31	0.286
Epilepsy	2.31 ± 1.23	2.20 ± 1.28	2.36 ± 1.21	0.203
Headache	2.75 ± 1.43	2.80 ± 1.43	2.73 ± 1.43	0.795
Multiple sclerosis	2.36 ± 1.27	2.32 ± 1.21	2.38 ± 1.30	0.826
Movement disorders	2.50 ± 1.24	2.28 ± 1.18	2.59 ± 1.25	0.093
Neuromuscular diseases	2.57 ± 1.37	2.66 ± 1.44	2.54 ± 1.34	0.636

## Discussion

This study aimed to assess the efficacy as well as the barriers and challenges of headache education. Data from 227 respondents were included. Neurology residents in the introductory program (R1) constituted 28.6% of the participants, whereas 71.4% of them were allocated to the main program. The majority of them (74.9%) received prior headache training.

More than half of residents self-rated their knowledge as good or very good for migraine (62.6%), tension-type headache (60.4%), and cluster headache (55.5%), whereas less than a half of residents self-rated their knowledge as good or very good for trigeminal post-traumatic headache (39.6%), autonomic cephalgias (40.5%), and medication overuse headache. The most commonly experienced challenges by the residents included difficult diagnosis (30.4%), treatment difficulties due to comorbidities (19.8%), and unclear medical histories. This is in line with a previous study where approximately half of the respondents reported that the diagnosis and treatment of patients with headaches were challenging. This is particularly of concern as the majority, estimated at 11%-20% of consultations, were related to headaches [10].

The most frequently reported barriers to optimal treatment of headache patients were the existence of challenges in collaboration between patient and therapist (24.7%) and difficulties in the diagnosis of headache patients (20.7%). However, correct diagnosis is the mainstay of clinical management of primary headache disorders and targeted educational interventions are needed. In a previous international survey of neurologists, explicit diagnostic criteria were only used in 56% of cases [14]. 

Similarly, the most common barriers reported by a study were connected to diagnosis and treatment. An unclear medical history is reported by more than half of residents as a difficulty, which may also overlap with comorbidities being reported as a common barrier [2]. This is troubling, as the diagnosis of headache disorders depends on the patient’s medical history. 

The proportion of headache consultations was 30% or higher among 17.4% of residents, which is higher compared to a previous study (10). Of note, only 8.3% of residents referred >30% of headache patients to specialized headache centers, and the most commonly reported reasons for referral to neurology were a suspicion of an underlying serious disease (31.7%) and diagnostic uncertainty (30.8%). 

Simple analgesics could potentially cause medication overuse, as perceived by 33.5% of residents, with no significant difference in residents’ perceptions between the introductory and main programs. In a previous study, opioids were expected to cause headaches (7). Over the years, the concept of medication overuse headaches has evolved, although the exact pathophysiology is still being investigated. It has to be differentiated from other secondary chronic headache disorders by a careful history, targeted examination, and details of medication intake [15].

Lack of healthcare providers and residents well educated in headache management, paucity of available headache prophylactic medications, and ease of obtaining over-the-counter analgesics for pain have combined to result in high rates of medication overuse headaches. In these settings, easy access to analgesics from private-sector pharmacies may provide temporary relief from headaches while lessening the incentive to seek an adequate medical assessment of their headaches - a pathway likely to increase the burden of headaches over time. Almost half of the residents stated that patients seek a non-pharmacological option for headache treatment once in a while (45.4%). If asked by the patient, the most commonly recommended non-pharmacological interventions were exercise (15.9%) and diet (14.1%).

The study was not without limitations. The study was limited by being a questionnaire, which inherently has the risk of recall, interviewer, and response bias. Another limitation is that the majority of the residents who participated are from R1 to R3, who are juniors, while the most knowledgeable residents, R4 and R5, did not participate very well. Therefore, this can have an impact on the results. An area of strength in this study is that the questionnaire used achieved a high response rate. A limited number of Saudi studies were published in this scope; thus, this study is considered a valuable base for evidence. Another strength of this study is that it included participants from variable demographical backgrounds and socio-economic statuses from different regions of Saudi Arabia, which would aid the authorities in dealing with the issue from all aspects. Evidence-based data regarding headaches should be displayed and spread through media platforms in the community. Also, educational campaigns should be held to increase awareness of this topic. More headache seminars and symposiums should be held to shed light on the headache education curriculum. Further research using a standardized approach to the common headache disorders with a pre-test and post-test opinion poll is recommended to gather evidence and reveal the standards of headache medicine understanding.

## Conclusions

It was found that headache issues were most frequently encountered among neurology residents due to challenging diagnosis and treatment difficulties brought on by comorbidities. The most often cited obstacle to providing headache patients with the best possible care was difficulties in patient-therapist collaboration. Utilizing the International Classification of Headache Disorders (ICHD) was more common than using Saudi guidelines for headache diagnosis and management. The most often reported causes for referral to neurology were suspicions of an underlying serious condition. Further research is recommended to gather additional evidence about this topic to identify gaps and overcome potential barriers in the current practice. Efforts should be made to overcome gaps in headache education during neurology residency training to facilitate a better educational process and appropriate patient care.
